# Cerebellar Atrophy and Changes in Cytokines Associated with the *CACNA1A* R583Q Mutation in a Russian Familial Hemiplegic Migraine Type 1 Family

**DOI:** 10.3389/fncel.2017.00263

**Published:** 2017-08-29

**Authors:** Svetlana F. Khaiboullina, Elena G. Mendelevich, Leyla H. Shigapova, Elena Shagimardanova, Guzel Gazizova, Alexey Nikitin, Ekaterina Martynova, Yuriy N. Davidyuk, Enver I. Bogdanov, Oleg Gusev, Arn M. J. M. van den Maagdenberg, Rashid A. Giniatullin, Albert A. Rizvanov

**Affiliations:** ^1^Department of Genetics, Institute of Fundamental Medicine and Biology, Kazan Federal University Kazan, Russia; ^2^Department of Neurology, Kazan State Medical University Kazan, Russia; ^3^Federal Research and Clinical Center, Federal Medical-Biological Agency of Russia Moscow, Russia; ^4^Innovation Center, RIKEN Yokohama, Japan; ^5^Preventive Medicine and Diagnosis Innovation Program, RIKEN Yokohama, Japan; ^6^Departments of Human Genetics and Neurology, Leiden University Medical Center Leiden, Netherlands; ^7^Department of Neurobiology, A. I. Virtanen Institute for Molecular Sciences, University of Eastern Finland Kuopio, Finland

**Keywords:** migraine, FHM1, cytokines, inflammation, nociception

## Abstract

**Background:** Immune mechanisms recently emerged as important contributors to migraine pathology with cytokines affecting neuronal excitation. Therefore, elucidating the profile of cytokines activated in various forms of migraine, including those with a known genetic cause, can help in diagnostic and therapeutic approaches.

**Methods:** Here we (i) performed exome sequencing to identify the causal gene mutation and (ii) measured, using Bio-Plex technology, 22 cytokines in serum of patients with familial migraine (two with hemiplegic migraine and two with migraine with aura) from a Russian family that ethnically belongs to the Tatar population. MRI scanning was used to assess cerebellar atrophy associated with migraine in mutation carriers.

**Results:** Whole-exome sequencing revealed the R583Q missense mutation in the *CACNA1A* gene in the two patients with hemiplegic migraine and cerebellar ataxia with atrophy, confirming a FHM1 disorder. Two further patients did not have the mutation and suffered from migraine with aura. Elevated serum levels of pro-inflammatory and pro-nociceptive IL-6 and IL-18 were found in all four patients (compared to a reference panel), whereas pro-apoptotic SCGF-β and TRAIL were higher only in the patients with the FHM1 mutation. Also, cytokines CXCL1, HGF, LIF, and MIF were found particularly high in the two mutation carriers, suggesting a possible role of vascular impairment and neuroinflammation in disease pathogenesis. Notably, some “algesic” cytokines, such as β-NGF and TNFβ, remained unchanged or even were down-regulated.

**Conclusion:** We present a detailed genetic, neurological, and biochemical characterization of a small Russian FHM1 family and revealed evidence for higher levels of specific cytokines in migraine patients that support migraine-associated neuroinflammation in the pathology of migraine.

## Introduction

Migraine is a common episodic brain disorder involving persistent changes in multiple body systems during attacks as well as the interictal period. Two main types of migraine exist: migraine with aura and migraine without aura (Headache Classification Committee of the International Headache Society (IHS), 2013). Familial hemiplegic migraine (FHM) is a rare monogenic type of migraine with aura for which several genes have been identified (van den Maagdenberg et al., 2007) and the underlying neuronal mechanisms are well-characterized (Ferrari et al., 2015).

Traditionally, much attention is given to a neuronal or vascular origin of migraine (Pietrobon and Moskowitz, 2013), however, evidence is accumulating that an aberrant immune response may also play a role in disease pathogenesis (Levy, 2009). Recently, it was suggested that a shifted profile of T-cells in migraine patients would point to a possible autoimmune nature of the disease (Arumugam and Parthasarathy, 2016). Furthermore, studies implicated several pro-inflammatory cytokines, such as IL-1 and IL-6, to be involved in migraine (Uzar et al., 2011; Zhang et al., 2012). These cytokines potentially target a wide range of cells including leukocytes, trigeminal nociceptors, and neuroglia to exert their effect on migraine pathophysiology (Zhang et al., 2012). Also, cytokines can promote the release of the peptide calcitonin gene-related peptide (CGRP) produced in neurons (Neeb et al., 2011), an important migraine neuroimmune mediator that further facilitates a migraine attack. It has been shown that CGRP, can stimulate the secretion of various cytokines by T-cells (Levite, 1998; Cuesta et al., 2002) suggesting a bidirectional crosstalk between cytokines and migraine-relevant neuropeptides. Notably, release of CGRP could contribute to increased cortical excitability (Tozzi et al., 2012) along with disrupted neurovascular coupling in migraine (Jacobs and Dussor, 2016).

Cytokines released by leukocytes are known to affect the function of neurons in the CNS and contribute to the regulation of the pain threshold. For example, headaches were documented after tumor necrosis factor-alpha (TNFα) injection (Schiller et al., 1991). Also, migraine was associated with elevated serum levels of TNFa and IL-1 (Perini et al., 2005). A study of cytokines in cerebrospinal fluid (CSF) of migraine patients revealed an increased level of IL-1, receptor antagonist (IL-1ra), monocyte chemoattractant protein-1 (MCP-1), and transforming growth factor-1 (TGF-1) (Bo et al., 2009). However, in contrast to other neurological diseases, such as multiple sclerosis (Khaibullin et al., 2017) or—more closely related to migraine—stroke (Chamorro et al., 2012), in which a neuroinflammatory component of disease pathology is well-known, our knowledge on the role of cytokines in migraine is limited. Changes in cytokine level in CSF found in neurodegenerative diseases such as multiple sclerosis suggested that cytokines could play role in managing brain damage and determine cortical excitability. Thus, microglia, the immune cells that reside in the brain and are the major source of pro-inflammatory cytokines, co-determine the development of cortical spreading depression (CSD) (Grinberg et al., 2013; Pusic et al., 2014), which is the electrophysiological correlate of the migraine aura. Studies in transgenic mouse models with human pathogenic FHM mutations (Ferrari et al., 2015) have highlighted specific genetic and neurobiological mechanisms that can explain the spectrum of clinical symptoms, such as the aura and the cerebellar ataxia seen with specific mutations, suggesting that FHM may also be an attractive condition to understanding better the contribution of the cytokines in migraine.

In this study, we present the profile of multiple cytokines along with a genetic and neurological characterization of a small FHM family from Russia. Whole-exome sequencing revealed a R583Q missense mutation in the *CACNA1A* gene in the two most affected persons of this family, i.e., those that had hemiplegic migraine and cerebellar atrophy and ataxia. The other two patients did not carry the mutation and were diagnosed as patients with migraine with aura. Migraine patients of this family have high levels of specific cytokines, some of which are high only in patients with the FHM1 mutation, supporting neuroinflammation in migraine possibly with a specific cytokine signature in those with hemiplegic migraine and cerebellar ataxia.

## Methods

### Subjects

Serum samples from four patients (two of which had a diagnosis of hemiplegic migraine, as confirmed by genetic testing, and two with migraine with aura), who belong to a Russian family of Tatar origin, and 11 unrelated healthy individuals of mixed ethnicity (that served as a reference sample) were collected. No serum was collected from non-migraine relatives. The Institutional Review Board of the Kazan Federal University approved the study and informed consent was obtained from each study subject according to the guidelines approved under this protocol (article 20, Federal Law “Protection of Health Right of Citizens of Russian Federation” N323-FZ, 11.21.2011). Written informed consent was obtained from the participants of this study.

### Multiplex analysis

Serum cytokine levels were analyzed using the Bio-Plex Pro Human Cytokine 21-plex Panel (Bio-Rad, Hercules, CA, USA), which is a multiplex magnetic bead-based antibody detection kit, following the manufacturer's instructions. Aliquots of serum (50 μL) from the four patients and 11 healthy controls were used. A minimum of 50 beads per analyte was acquired. Median fluorescence intensities were collected using a Luminex 200 analyzer (Luminex, Austin, TX, USA). Data collected was analyzed using MasterPlex CT control software and MasterPlex QT analysis software (MiraBio, San Bruno, CA, USA). Standard curves for each analyte were generated using standards provided by manufacturer.

### ELISA

Interleukin-6 (IL-6) levels were determined in serum (100 μL) using an ELISA kit (Vektor best, Novosibirsk, Russia) according to the manufacturer's recommendations.

### Whole-exome sequencing

Blood samples were collected from the four patients of the family (patients F, D, S, and B) and used for DNA extraction (Qiagen, GmbH, Germany). DNA concentration was quantified using a Qubit double-stranded DNA High Sensitivity Assay Kit and Qubit fluorometer (ThermoFisher Scientific, MA, USA). Some 4 ng/μL of genomic DNA in a total volume of 100 μL was sheared to an average fragment size of 200 bps using a Q800R sonicator (QSonica, Newtown, USA). The size distribution of the fragmented DNA was assessed on a Bioanalyzer High Sensitivity DNA chip (Agilent Technologies, Santa Clara, USA). In total, 25 μL of sheared DNA was used as input for library preparation by KAPA Library Preparation Kit (Kapa Biosystems, Woburn, MA, USA) according to manufacturer's instructions. Resulting libraries were combined into pools of six for exome enrichment by means of NimbleGen EzCap Human v3.0 Exome Enrichment Kit (Roche NimbleGen Inc, Basel, Switzerland). Captured libraries were assessed for quality using the Agilent High Sensitivity DNA assay and yield using Qubit double-stranded DNA High Sensitivity Assay Kit and Qubit fluorometer (ThermoFisher Scientific, MA, USA). The pull of four samples per flow cell lane was sequenced using the Illumina HiSeq2500 platform and version 3 SBS chemistry to generate 100-bp paired-end reads (2x100PE). The Illumina bcl2fastq2 Conversion Software v2.17 was used for de-multiplexing.

Sequence data was aligned to build hg38 (released Dec 2013) of the human genome using the Novoalign alignment tool (V2.08.11); sequence alignment files were converted using SAMtools (v0.1.16) and Picard tools (v1.42). The adapter sequences and low quality reads was trimmed using Cutadapt and Trimmomatic, reads mapped to a reference genome sequence. (GRch37/hg19) using the algorithm BWA-MEM (v0.7.15), quality control of input data, alignment, concentration, and coverage of target regions was carried out by FastQC, BAMQC, and NGSrich, duplicates was removed by sambamba (v0.6.5). The nucleotide variation was searched with GATK HaplotypeCaller + UnifiedGenotyper (to produce a combined VCF-file).

Annotation was performed with SnpEff (analysis of all transcripts), ANNOVAR (allele frequency analysis ExAC, 1000G and ESP6500, algorithms the functional significance SIFT, PolyPhen2, Mutation Taster, FATMM, CADD, DANN, Eigen), and Alamut Batch (effect on splicing, database dbSNP, ClinVar, HGMD Professional). Raw sequencing data and the mapping results are available upon request.

### Magnetic resonance imaging

Magnetic resonance imaging (MRI) study was carried out with ExcelartVantage 1.5T device (Toshiba, Japan). Standard T1 and T2 modes of the weighted imaging were used.

## Results

### Family history

**Figure 1:**
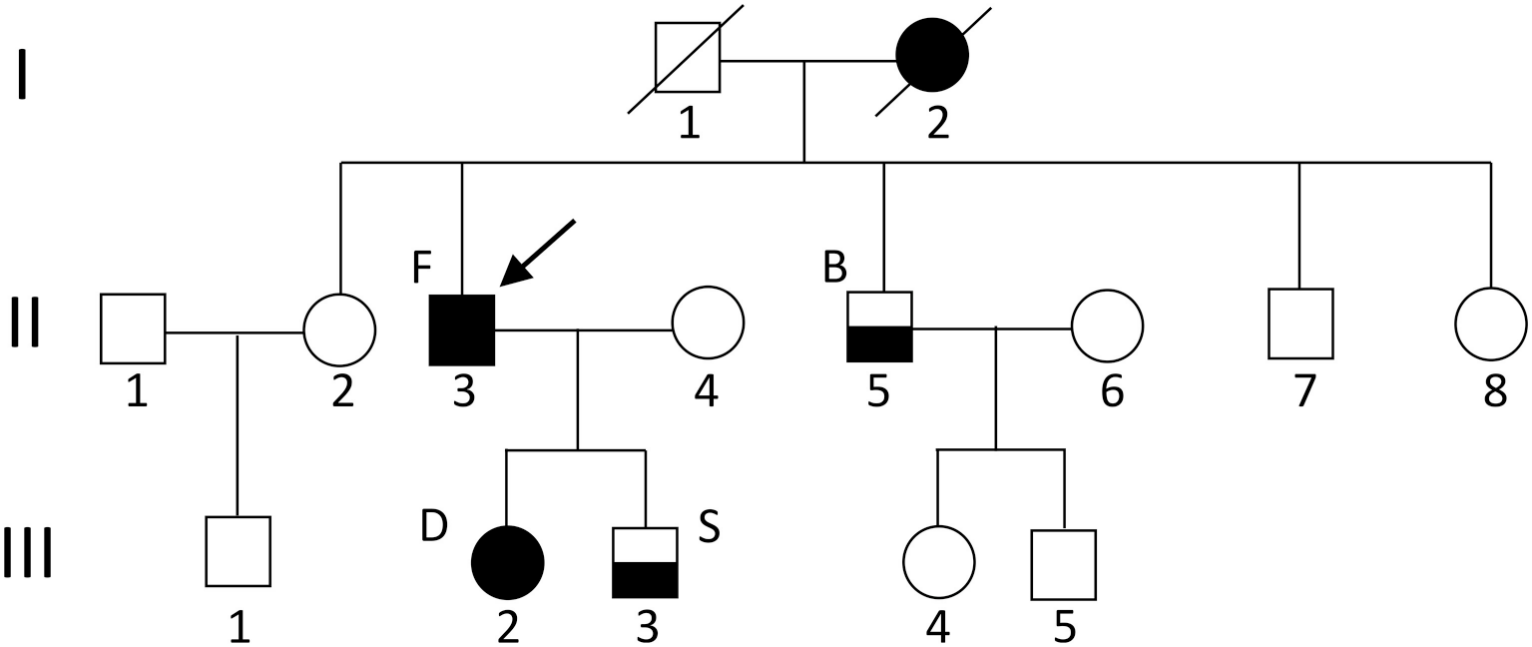
Pedigree of the family. The arrow indicates the proband. Circles indicate females; squares indicate males. The black square/circle indicates a carrier of the FHM1 R583Q mutation with hemiplegic migraine. Lower black half indicates a diagnosis of migraine with aura and no mutation. No DNA was available for genetic testing in other family members.

In our Russian (ethnic Tatars) family of 13 individuals, four members have migraine (Figure 1). Proband patient F (II.3) (in the figure indicated with an arrow) has hemiplegic migraine as does his daughter [patient D (III.2)]. The son [patient S (III.3)] and the brother [patient B (II.5)] of the proband have migraine with aura. A detailed description of the clinical characteristics is provided below. The proband's mother anecdotally suffered from severe headaches but it was not possible to obtain a detailed clinical description of her.

### Patients description

A detailed analysis including neurological and instrumental examination was available for patients F and D.

#### Patient F (father), 41-years-old at diagnosis

**Figure 2:**
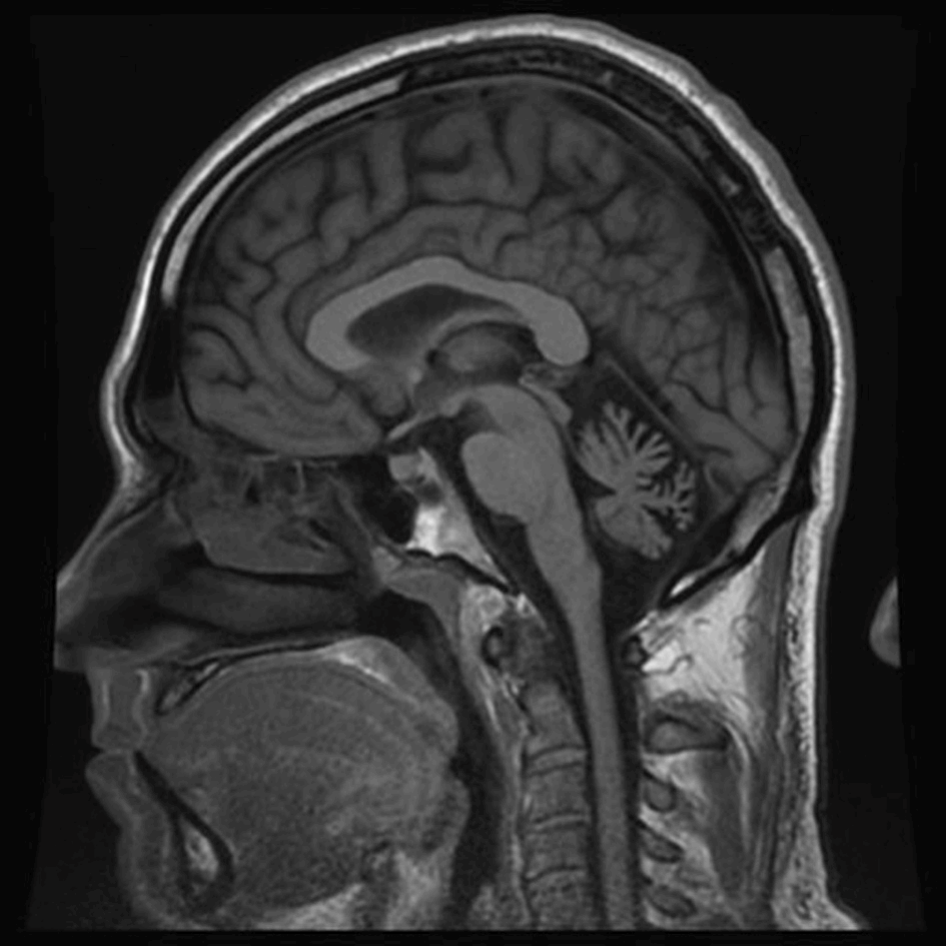
MRI examination (T1 weighted sagittal image) of patient F. Notice severe cerebellar atrophy.

From the age of 13, patient F complains about headaches preceded by weakness of arm and leg with some speech impairment. Headaches usually present on one side, often last several hours, and are accompanied by nausea and intolerance to light and sound. Migraine attacks appear 2–3 times per year. From a young age, the patient is having ataxia-like symptoms, which he links to multiple traumatic brain injuries in childhood. Neurological examination revealed absence of apparent pathology in cranial nerves, and no changes in muscle tone or strength in the limbs during the interictal period. Tendon reflexes are normal and symmetrical, however, slight adiadochokinesia with prevalence on the right side as well as hypermetria on the right side of the heel-knee test is detected. Also, instability in the Romberg test is detected. Tactile sensitivity (superficial and deep) is not affected. The patient does not have cognitive impairment. MRI examination revealed a reduced size of the cerebellum (Figure 2).

#### Patient D (daughter), 18-years-old at diagnosis

**Figure 3:**
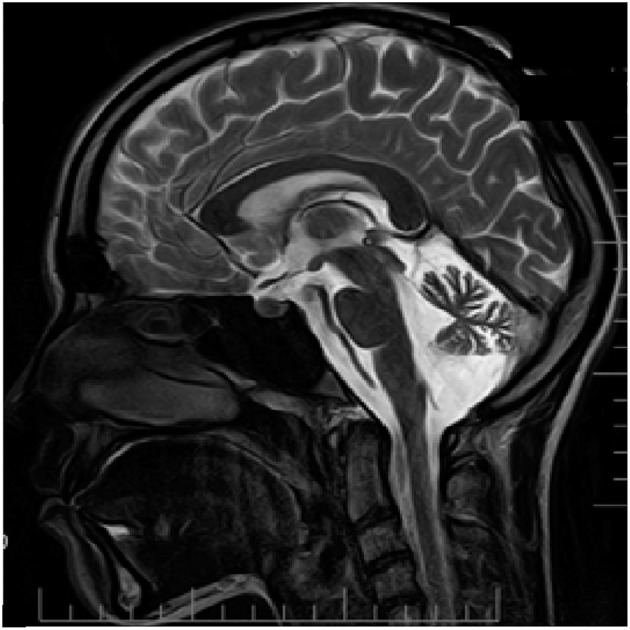
MRI examination (T2 weighted sagittal image) of patient D. Notice clear cerebellar atrophy. Patient information and technical text were closed.

From the age of 12, patient D has headaches preceded by an aura that includes weakness and numbness in the right limbs along with speech impairment that lasts about 15–20 min. Headache was typically localized to the left temporal region of the head and associated with photo- and phonophobia, nausea and occasional vomiting. The frequency of the headaches is once every 3–4 months; duration of the headaches was several hours. The patient recalls first appearance of her attacks after multiple mild traumatic brain injuries. Neurological examination reveals minor signs of adiadochokinesia (right > left) and heel-knee probe which is slightly asymmetrical with hypermetria on the right side. Tactile sensitivity was normal. No cognitive impairments were found. Like seen in her father (patient F), MRI examination revealed essential cerebellar atrophy (Figure 3).

#### Patient B (brother), 35-years-old at diagnosis

The patient B most likely has attacks of migraine with aura and speech impairments. More recently, symptoms of numbness and weakness of the upper extremity, the low part of the face and the tongue with difficulties of articulation lasting for 30–40 min occurred without subsequent headaches.

#### Patient S (son), 17-years-old at diagnosis

**Figure 4:**
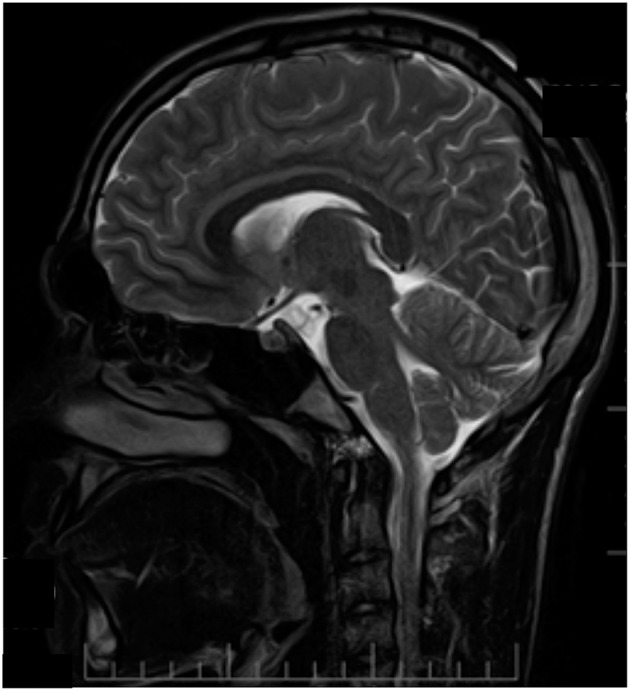
MRI examination (T2 weighted sagittal image) of patient S. No signs of atrophy in cerebellum. Patient information and technical text were closed.

At the age of 14, patient S had a single pulsation headache attack, preceded by weakness, and half-body numbness. Later in life, migraine attacks with visual aura were reported. Notably, unlike his father and sister, MRI examination was unremarkable (Figure 4).

### Genetic analysis

All four patients of the family were subjected to whole exome analysis (see Methods). We identified a pathogenic p.Arg583Gln (NM_023035.2:c.1748G>A) mutation that is the cause of hemiplegic migraine in patients F and D. Patients B and S did not have the mutation. Segregation of the mutant *CACNA1A* allele in the family could be assessed using the C-terminal CAG expansion (expansions of this repeat above ~20 repeats are known to cause spinocerebellar ataxia type 6; Zhuchenko et al., 1997) as a marker. Notably, the mutant allele (which had 12 CAG repeats) found in patients D and F was not present in patients B or S, who had 11 and 13 and 7 and 13 CAG repeats, respectively; so all CAG expansions are non-pathogenic. In line with available information, patients B and S are diagnosed with migraine with aura.

### Cytokine profile

A total of 22 cytokines were analyzed in serum of four patients of the family and a set of 11 unrelated healthy controls that served as a reference. Although the small number of migraine patients with or without an FHM1 mutation did not allow for a statistical analysis, a comparison revealed higher levels of several cytokines in FHM1 and/or migraine with aura cases. Of special interest were cytokines that were similarly affected in both mutation carriers (patients F and D) but not changed in migraine with aura relatives or the controls. First, we found that serum levels of classical pro-inflammatory IL-6 and IL-18 were higher in all patients with migraine, irrespective of the presence of the mutation (Table 1). In contrast, levels of SCGF-β, MIF, and TRAIL were higher only in patients with the FHM1 mutation. Cytokines CXCL1, HGF, and LIF appear to be also higher in at least one patient with migraine with aura (Table 1). Cytokine IL-2Ra was up-regulated in only one of the mutation carriers (patient D). The level of IL-1a, IL-12(p40), chemokine C-C-motif ligand-7 (CCL7), CXCL12, and stem cell factor (SCF) did not differ between patients and controls, whereas IL-3, macrophage colony-stimulating factor (M-CSF), interferon-alpha (IFNα) and TNFβ in all patients were particularly low, in most cases at the detection limit. The cytokines with higher levels could be divided into three groups: pro-inflammatory and pro-nociceptive (IL-6, IL-18, CXCL1, MIF) and promoting angiogenesis (HGF, SCGF-β). Up-regulation of LIF, a mast cell growth-enhancing factor, in mutation carriers further supports the role of mast cells in migraine pathogenesis.

**Table 1:** Serum cytokines in patients diagnosed with migraine.

**Analyte**	**Controls**	**Patient F (FHM1 R583Q)**	**Patient D (FHM1 R583Q)**	**Patient S (MA and no R583Q mutation)**	**Patient B (MA and no R583Q mutation)**
IL-1a	0.8 ± 0.4	0.5	0.5	0.2	0.4
IL-2RA	64.9 ± 22.1	50.1	202.6	28.8	25.4
IL-3	12.4 ± 2.1	2.3	2.3	5.4	3.5
IL-6	89.89 ± 4.55	212.2	200.9	193.9	212.7
IL-12(p40)	92.4 ± 12.5	85.3	89.2	89.2	93.2
IL-16	101.03 ± 25.9	79.8	171.3	45.3	34.6
IL-18	11.0 ± 2.6	47.7	125.2	22.9	37.6
CCL7	18.3 ± 2.1	15.8	20.3	17.2	18.2
CCL27	436.9 ± 51.6	613.5	684.9	429.1	377.2
CXCL1	39.6 ± 11.9	129.6	171.5	34.6	230.5
CXCL9	669.7 ± 182.1	2318.9	945.7	384.6	735.7
CXCL12	219.2 ± 135.2	88.5	164.7	79.5	87.5
HGF	221.6 ± 45.5	790.9	1052.1	280.5	572.9
IFNα	22.2 ± 1.8	1.2	2.3	2.4	6.4
LIF	4.5 ± 1.2	16.2	29.3	1.3	10.5
M-CSF	3.2 ± 0.4	4.2	3.2	4.3	4.2
MIF	945.7 ± 319.9	2980.9	4112.0	120.3	1404.3
β-NGF	31.1 ± 21.6	11.7	9.3	7.9	8.6
SCF	139.6 ± 35.9	168.9	156.6	103.8	155.0
SCGF-β	446.4 ± 159.2	2495.1	16229.8	758.4	877.5
TNFβ	0.9 ± 0.1	0.2	0.2	0.2	0.3
TRAIL	56.9 ± 17.6	241.6	123.0	12.3	24.2

*FHM1 R583Q: familial hemiplegic migraine type 1 carrier of the CACNA1A R583Q mutation; MA, migraine with aura (no carrier of the CACNA1A R583Q mutation)*.

## Discussion

Here we present a small Russian FHM1 family with two patients (D and F) who carry the R583Q missense mutation in the *CACNA1A* gene. Both patients suffered from hemiplegic migraine with aura and showed neurological cerebellar changes with cerebellar atrophy, as supported by neuroimaging.

The R583Q mutation is a previously described pathogenic mutation, often (Ducros et al., 2001), but not in all cases (Terwindt et al., 2002), associated with cerebellar ataxia, and therefore the likely cause of disease in patients D and F. The mutation affects the voltage sensor of the S4 helix of the α_1A_ subunit of voltage-gated Ca_V_2.1 (P/Q-type) Ca^2+^ channels and results in a shift of the voltage dependence of activation and inactivation to more negative potentials with a slower recovery from inactivation (Kraus et al., 2000). Given the highest level of expression of Ca_V_2.1 channels in cerebellum (Fletcher et al., 1996) it is long recognized that the gain-of-function effect of R583Q-mutated channels can explain cerebellar atrophy and cerebellar ataxia in patients. The link with various forms of ataxia and mutations in *CACNA1A* is rather complex, suggesting that neuronal calcium influx through Ca_V_2.1 channels is tightly controlled. Notably, some, but not all, gain-of-function FHM1 mutations are associated with chronic cerebellar ataxia (Ferrari et al., 2015), whereas loss-of-function mutations in the same gene, exerting dominant-negative effects (Jeng et al., 2006; Dorgans et al., 2017), cause episodic ataxia type 2 (EA2) (Ophoff et al., 1996). Finally, specific C-terminal CAG expansions, with complex functional effects involving e.g., the aggregation of alternative spliced C-terminal fragments (Du et al., 2013; Mark et al., 2015), cause late-onset cerebellar ataxia, namely spinocerebellar ataxia type 6 (SCA6) (Zhuchenko et al., 1997; Giunti et al., 2015).

A novelty of the study was the extensive profiling of multiple cytokines in migraine patients, including those with an FHM1 gene mutation. One of key questions is whether dysregulation of cytokines could directly or indirectly contribute to the main pathological changes in our patients including the hemiplegic migraine and the cerebellar atrophy that is linked with the ataxia.

We found an increased serum levels or IL-6 in both FHM1 patients (D and F), as well as in the two migraine with aura patients without the genetic mutation (B and S). IL-6 is an abundant prototype inflammatory cytokine, shown to be elevated in patients with the common forms of migraine (Uzar et al., 2011; Zhang et al., 2012). In animal experiments, this cytokine can enhance excitability of dural afferent nerves and produce migraine-related allodynia (Yan et al., 2012). Along with IL-6, pro-inflammatory cytokine IL-18 is also up-regulated in serum of all migraine patients, albeit stronger in patients with the gene mutation. Consistent with the higher level of IL-6, which is a strong activator of CXCL1 (Roy et al., 2012), we found high levels of CXCL1 in all but one (patient S) migraine patients. CXCL1, like IL-6 is a pro-inflammatory and pro-nociceptive cytokine (Wang et al., 2008; Yang et al., 2016) that is co-expressed in sensory neurons together with neuropeptide CGRP (Cao et al., 2016), which is a major migraine mediator (Tfelt-Hansen and Olesen, 2011).

High serum levels of LIF and MIF were consistently found in FHM1 patients (but not in patients without the gene mutation). LIF is a member of the IL-6 family of cytokines, which can act directly on small-size nociceptive trigeminal neurons (Tamura et al., 2003). LIF also promotes the growth of mast cells (Hiragun et al., 2000; Gyotoku et al., 2001), which are considered to play a triggering role in pathogenesis of migraine (Levy et al., 2006; Kilinc et al., 2017). MIF is a pro-inflammatory cytokine expressed in the nociceptive system in neurons and glial cells that may maintain inflammatory states (Vera and Meyer-Siegler, 2003). Thus, combined pro-nociceptive and pro-inflammatory action of cytokines IL-6, IL-18, CXCL1, LIF, and MIF could occur in the trigeminovascular system, which comprises of peripheral trigeminal neurons and vessels in meninges located outside the brain blood barrier, that might explain specific migraine related changes in our patients. Previous studies in experimental animal studies suggested a possible role of enhanced neuronal excitability in the brain of carriers of an FHM1 gene mutation (van den Maagdenberg et al., 2004; Nair et al., 2010; Pietrobon and Moskowitz, 2013). Our current study extends this view suggesting the influence of pro-inflammatory cytokines on enhanced neuronal excitability and, possibly, aura features in patients.

The observed pronounced up-regulation seen with certain cytokines in the two FHM1 patients may reveal a contribution to the enhanced neuronal hyperexcitably and cerebellar atrophy in FHM1 (Ferrari et al., 2015). For instance, pro-apoptotic TRAIL (TNF-related apoptosis-inducing ligand) can induce degeneration of neurons (Kichev et al., 2014) whereas down-regulation of TRAIL itself and of its death receptors with specific antibodies ameliorates neuronal damage (Cantarella et al., 2014). However, it cannot be excluded that abnormal cytokine levels may in fact reflect some secondary effect, for instance they may occur as consequence of long-lasting multiple pathological changes and/or recurrent pain attacks in patients D and F.

A major limitation of the study is the very small number of investigated patients; too small even to apply statistics. Large FHM families are difficult to obtain—certainly in a country the size of Russia with patients spread over a large geographical distance—and this study has its merits as hypothesis-generating. Our study suggests previously not recognized associations of cytokines in migraine pathophysiology. Whether these cytokines actually play a role should be assessed in larger studies. Any neuroinflammatory role of such abnormal cytokines may be investigated in experimental animal models.

Genome-wide association studies that systematically test for association between single nucleotide polymorphisms (SNPs) (as they also occur in cytokine-encoding genes) and a phenotype have not surfaced cytokine genes among the top migraine hits in the largest migraine GWAS (that consisted of over 59,000 patients) (Gormley et al., 2016). Still, a genome-wide analysis of blood gene expression in 83 migraine patients (and a similar number of controls) for which data of close to 18,000 expression probes were measured, although they did not detect a genome-wide significant hit for a cytokine at the individual gene level, strongly suggests the relevance of cytokines in migraine at the pathway level, as highlighted by significant hits with relevant canonical pathways that include “immune system,” “interferon signaling,” and “cytokine signaling in immune system” (Gerring et al., 2017). Lack of finding a significant association for a cytokine at the SNP level is perhaps not surprising given that a GWAS study that investigated the genetic underpinning of concentrations of 41 circulating cytokines (of those only SCGF-β, IL-18, TRAIL, HGF, Il-2ra, IL-16, MIF were investigated in our study) in whole blood only 27 genome-wide significant loci were identified, which for all loci explained only very little of the variance (Ahola-Olli et al., 2017), so many (non-)genetic factors that co-determine circulating levels of these cytokines remain unknown.

Collectively, the serum cytokine profile identified in two patients with genetically confirmed FHM1 suggests novel contributors to main disturbances implicated in the pathogenesis of this type of migraine. High levels of certain pro-apoptotic cytokines may have contributed to the cerebellar degeneration seen in the mutation carriers, whereas a concerted up-regulation of pro-nociceptive cytokines may reflect more migraine as phenotype. These results suggest that combined genetic and non-genetic neuroimmune mechanisms could be involved in the complex clinical phenotype of monogenic and other types of migraine.

## Author contributions

SK: writing the manuscript, specifically the cytokine data analysis, and discussion; intellectual contribution into the discussion; EGM: Selection and recruitment of patients; collection and interpretation of clinical data, and writing the manuscript; LS and GG: whole-exome sequencing, data analysis; ES: study design, exome data analysis; AN: collecting and preliminary analysis of whole exome data; EM: Luminex analysis of cytokine levels; YD: isolation of genomic DNA from patient samples; Quality control and preparation for molecular genetics analysis; EB: collection and interpretation of clinical data; OG: analysis of whole-exome data, wrote the manuscript; AV and RG: design of the study, writing the manuscript; AR: Design of molecular genetics and cytokine profiling experiments. Writing the manuscript.

### Conflict of interest statement

The authors declare that the research was conducted in the absence of any commercial or financial relationships that could be construed as a potential conflict of interest.

## Abbreviations

IL-1a: Interleukin-1a
IL-2Ra: Interleukin-2Ra
IL-3: Interleukin-3
IL-6: Interleukin-6
IL-12p40: Interleukin-12 subunit p40
IL-16: Interleukin-16
IL-18: Interleukin-18
CCL7: C-C motif ligand 7
CCL27: C-C motif ligand 27
CXCL1: C-X-C-motif ligand 1
CXCL9: C-X-C motif ligand 9
CXCL12: C-X-C motif ligand 12
HGF: Hepatocyte growth factor
IFN-α2: Interferon alpha 2
LIF: Leukemia inhibitory factor
M-CSF: Macrophage colony-stimulating factor
MIF: Macrophage migration inhibitory factor
β-NGF: beta-nerve growth factor
SCF: Stem cell factor
SCGF-β: Stem cell growth factor beta
TNF-β: Tumor necrosis factor-beta
TRAIL: Tumor necrosis factor-related apoptosis-inducing ligand.
